# F.E.M. Stress-Investigation of Scolios Apex

**DOI:** 10.2174/1874120701812010051

**Published:** 2018-07-31

**Authors:** A. Daghighi, H. Tropp, N. Dahlström, A. Klarbring

**Affiliations:** 1 Department of Clinical and Experimental Medicine, Linköping University, 581 83 Linköping, Sweden; 2 Department of Clinical and Experimental Medicine & Division of Surgery Orthopedics and Oncology, Linköping University, 581 83 Linköping, Sweden; 3 Center for Medical Image Science and Visualization, Linköping University, 581 83 Linköping, Sweden; 4 Department of Management and Engineering, Division of Solid Mechanics, Linköping University, 581 83 Linköping, Sweden

**Keywords:** Scoliosis, FEM Stress-Investigation, Thoracal Idiopathic, Pathological mechanisms, Mechanical loading, Comsol model

## Abstract

**Background:**

In scoliosis, kypholordos and wedge properties of the vertebrae should be involved in determining how stress is distributed in the vertebral column. The impact is logically expected to be maximal at the apex.

**Aim:**

To introduce an algorithm for constructing artificial geometric models of the vertebral column from DICOM stacks, with the ultimate aim to obtain a formalized way to create simplistic models, which enhance and focus on wedge properties and relative tilting.

**Material/Methods:**

**** Our procedure requires parameter extraction from DICOM image-stacks (with PACS,IDS-7), mechanical FEM-modelling (with Matlab and Comsol). As a test implementation, models were constructed for five patients with thoracal idiopathic scoliosis with varying apex rotation. For a selection of load states, we calculated a response variable which is based upon distortion energy.

**Results:**

For the test implementation, pairwise t-tests show that our response variable is non-trivial and that it is chiefly sensitive to the transversal stresses (transversal stresses where of main interest to us, as opposed to the case of additional shear stresses, due to the lack of explicit surrounding tissue and ligaments in our model). Also, a pairwise t-test did not show a difference (n = 25, p-value≈0.084) between the cases of isotropic and orthotropic material modeling.

**Conclusion:**

A step-by-step description is given for a procedure of constructing artificial geometric models from chest CT DICOM-stacks, such that the models are appropriate for semi-global stress-analysis, where the focus is on the wedge properties and relative tilting. The method is inappropriate for analyses where the local roughness and irregularities of surfaces are wanted features. A test application hints that one particular load state possibly has a high correlation to a certain response variable (based upon distortion energy distribution on a surface of the apex), however, the number of patients is too small to draw any statistical conclusions.

## 1 BACKGROUND AND AIM

This paper concerns idiopathic scoliosis in adolescents. About 80 percent of structural scoliosis is classified as idiopathic scoliosis (American Association of Neurological Surgeons [1]). If there is an underlying known diagnosis, believed to be the chief causing factor, then the scoliosis is usually not classified as idiopathic, an example of this is neuromuscular scoliosis. The pathological mechanisms of scoliosis are multi-factorial and obviously in the idiopathic case not yet fully understood. Main factors that are recognized as essential are rotational lordosis with a growth-discrepancy between the anterior and posterior, asymmetrical growth and muscle activities, see Hefti [2]. Brink *et al*. [3] proposed that the anterior overgrowth is the result of the scoliotic mechanism. For deeper current knowledge about idiopathic scoliosis see *e.g.* the textbook by Newton *et al*. [4]. Using FEM-analysis based on models obtained from medical imaging, allows for noninvasive investigation of how stress is distributed at and near the apex, in scoliosis patients. Mechanical loading influences the rate of long bones and vertebrae and clearly altered loading is implicated in scoliosis (and other skeletal deformities), see *e.g.* Stokes [5]. This together with the aforementioned observations of growth-discrepancy between the anterior and posterior in scoliosis makes it interesting to gather knowledge about how stress from applied axial and shear forces respectively, is distributed in the vertebral column at, and near the apex, for varied severity of scoliosis.

### 1.1 Primary Aim:

To find a semi-global approach to simplistic, but rigorously defined, modelling that keep the main wedge and relative tilt features of the vertebrae. The requirement of rigour and simplicity together implies, in practice, that small local surface variations will be discarded and instead basic geometric shapes will be used.

### 1.2 Secondary Aim:

To implement the procedure from our primary aim in a test study that arises given a certain biomechanical hypothesis (see below). In order to describe the test study and the hypothesis which underlies it, we need some prerequisites and definitions, in particular, we need to define our response variable.

## 2 RESPONSE VARIABLE AND HYPOTHESIS

### 2.1 Background

 Our FEM (Finite Element Method) simulations were performed using Comsol Multiphysics (the successor of FEMLAB). Comsol has recently appeared in the research of the spine, *e.g.* for simulating a segment of the vertebral column in order to study if the stress-strain on the vertebral isthmus is affected by a bifid arc, see Quah *et al*. [6]. Many other competing FEM software exist, *e.g.* ANSYS, used by Anburajan & Divya [7], to simulate lumbar spine. Other examples can be found in Aleti *et al*. [8], where they use fundamental geometric figures to model the spine and among other things calculate the effect of many repetitions of certain stresses, Nédli *et al*. [9], where they study the compression of discs, upon applied stress to the vertebrae, and Shazly *et al*. [10] & [11], where they model vascular blood flow and study the changes with respect to spinal chord compression. Our model construction, however, starts from DICOM stacks (underlying chest CT-scans). From this, one can create CAD-like mesh models* via *segmentations, and then simulate load-states and measure some appropriate output or response variable. Our chosen response variable in the present FEM study is influenced by the software chosen, *i.e.* Comsol. Let us now present the response variable.

### 2.2 Response Variable

First note that an applied vertical downward stress on a vertebra above the apex will render a distortion energy response, at each point on a surface that is approximately parallel to the top or bottom surface of a model of the apex. The response will, of course, look different depending upon where the stress is applied (*i.e.* which load-state) is used, see Definition 2.1) on the one hand and upon the curvature, wedge and tilting properties of the vertebrae on the other hand. We have chosen to perform our analysis on artificial geometric models of the apex whose interface with the intervertebral discs are modeled as planar. Our artificial geometric models have planar interface with the intervertebral discs. For our test implementation on such models, the following definitions will be used.

#### 2.2.1 Definition (Load-State).

We define a load-state (in Comsol) to be a fixed set of specified solids in a Comsol model, together with non-void body load conditions and non-void fixed constraints. (In particular, given a load-state, the minimal conditions are fulfilled, for being able to simulate point-wise distortion energy calculations in a solid mechanics environment in Comsol).

#### 2.2.2 Definition (Focus Point).

Given a bounded planar domain, with a given coordinate grid and with pointwise calculated, non-constant values of distortion energy, for some model and a given load-state; assume that the global maximum occurs on the union of finitely many subdomains, curves and points. In the case where the global maximum is attained on a subdomain, we assign to that domain the point which constitutes the relative center of mass of the subdomain. If there is at least one subdomain, we use the point assigned to the subdomain of largest measure, with that property. That will be called the focus point. If there are several domains of the same measure the mean of their centers of mass is used. (Practical note: In cases when there is more than one subdomain and where we suspected that one of them could be the result of artefacts or inconsistencies (*e.g.* sharp edges) between the artificial model and the CT, manual investigation was performed and the focus point was decided ad hoc by the investigator)

#### 2.2.3 Definition (Apex Top Response Angle)

Fig. (**1**). Consider a 3D model of a vertebral column, from an upper body CT, and let Λ be a plane intersecting the apex in the model. Assume a load-state is given. Let ℓ be the projection, on Λ, of the line (as described in Section 3.2), that describes the tilting and rotation of the hips relative to the scanner bed in the CT. The apex top response angle with respect to Λ, for the given load-state, is the angle relative to ℓ, of a line passing through (1) the relative center of mass, of the closure of the intersection of the apex with Λ, and (2) a point at which the amplitude of the distortion energy is maximal (the focus point or a point whose properties best resembles such a point, chosen ad hoc by the investigator). When the load-state, Λ and ℓ are clear from the context, we shall simply use the term *apex top response angle*.

In this paper, we shall only apply the term apex top response angle to the situation of an artificial geometric model, where the top face of the vertebral body in the model is planar, and , Λ in Definition 2.3, will always be assumed to contain the top face, hence we shall be working in a situation that resembles the one depicted in Fig. (**1**).

Now that we have defined our response variable we can formulate a hypothesis that motivates the implementation describes above as our secondary aim.

#### 2.2.4 Hypothesis:

The wedge and tilting properties of the apex and nearby vertebrae, in scoliosis patients, render (for some appropriately chosen load-state) a distortion energy response, which accumulates in amplitude at an apex top response angle that varies as an analytic function of the apex vertebral rotation (for some interval).

#### 2.2.5 Remark

In order for our response variable to be logical, we need to verify that there are notable variations with respect to some common load-states and patient models. In order to best suit our practical purposes we devised it to be less sensitive to shear stresses, than to transversal, almost vertical (for a standing patient) stresses. Additional shear stresses were of less interest is due to the lack of explicit surrounding tissue and ligaments in our model. To assess the latter we investigated if there was a difference between the focii of the distortion energy response when comparing load states with additional left and right shear component respectively. An additional question to address is whether the chosen response variable was affected by interchanging orthotropic material parameters with isotropic material parameters.

## 3 METHOD

### 3.1 Overview

- Our suggested algorithm for creating artificial geometric models starts from DICOM stacks (see Section 3.6) and for our test implementation we only require models including the apex together with the upper and lower adjacent vertebrae.
- The test implementation involved creating, for each of 5 patients with idiopathic thoracic scoliosis (where the patients were chosen to have a variety of apex rotation values), two parallel models, one using isotropic material parameters and the other using orthotropic material parameters.
- For each isotropic model, we simulated 5 basic load-states (using a boundary load applied at the top face in the negative z-direction). For comparison purposes, we also simulated adjusted states associated to each of the basic states: one variant which included additional clockwise shear load components on the upper vertebra and one variant which instead included additional counter-clockwise shear load components on the upper vertebra. For each simulated load-state we calculated, using Comsol, the apex response angle.
- We used paired t-test in order to compare: (i) The 25 different pairs of isotropic and orthotropic based model simulations, respectively, of the 5 load-states without additional shear load components. (ii) The 25 pairs of load states, for the isotropic models, which had additional clockwise- and counter-clockwise-shear load components, respectively. (ii) The 25 pairs of basic load-states, for the isotropic models.

We calculated, for each of the basic load-states (LS), the sample correlation,

(1) $$\rho^{\mathrm{LS}}:=\frac{\sum_{j}\left(A_{j}^{\mathrm{LS}}-\bar{A_{j}^{\mathrm{LS}}})(R_{j}^{\mathrm{LS}}-\bar{R_{j}^{\mathrm{LS}}}\right)}{(\sum_{j}(A_{j}^{\mathrm{LS}}-\bar{A_{j}^{\mathrm{LS}}}{)}^{2}{)}^{\frac{1}{2}}(\sum_{j}(R_{j}^{\mathrm{LS}}-\bar{R_{j}^{\mathrm{LS}}}{)}^{2}{)}^{\frac{1}{2}}},$$

between the vector $A^{\mathrm{LS}}=(A_{1}^{\mathrm{LS}},...,A_{5}^{\mathrm{LS}})$ , of apex rotations and the vector, $R^{\mathrm{LS}}=(R_{1}^{\mathrm{LS}},...,R_{5}^{\mathrm{LS}})$ , of the response angles for the isotropic models with the given load-state (here $\bar{X}$ denotes the mean of a given vector X). We investigated whether or not a correlation was probable, and if so, whether or not that was more prominent for a particular load-state.

- Our secondary purpose was that of investigation of methodology. We created, for a single patient, 3 pairs of solid models, where each pair consisted of one with isotropic parameters and one with orthotropic parameters. The 3 types of models used where: an artificial geometric model, a model going from directly from manual segmentation (from CT-DICOM stacks)* via *minimal repairing to solid, and finally a segmentation model that was subdued harmonic smoothing before being imported as a solid into Comsol.

We point out that the main component of our work is the actual introduction of the algorithm for constructing artificial geometric models from MPR (multi-plane reconstruction) based on CT-scans. This yields a formalized method to obtain mechanically well-adapted models, which enhance and focus on certain mechanical responses which otherwise would be diminished due to small surface variations and also render shorter meshing and calculation times in FEM-analyses.

For the readers convenience we have collected in the Appendix, the details regarding distortion energy, material parameters and assignment of material properties for the orthotropic models, in practice.

#### 3.1.1 Scanning Device Details

 All chest CT were preoperative. The scanning device was SOMATOM Definition Flash, Siemens Medical Solutions, Forchheim, Germany. The CT used 4 mm intervals. The associated software for analyzing DICOM, was Sectra, IDS7, including the application MPR.

### 3.2 Measuring the Rotation of the Apex Vertebra and Sacrum to Table Angle

We did this manually, based upon the method of Aaro-Dahlborn [12], with the requirement that the initial chosen line in the method pass the vertebral groove (for alternative methods/representatives of apex axial rotation see *e.g.* Lam *et al*. [13]).

**Fig. (2):**
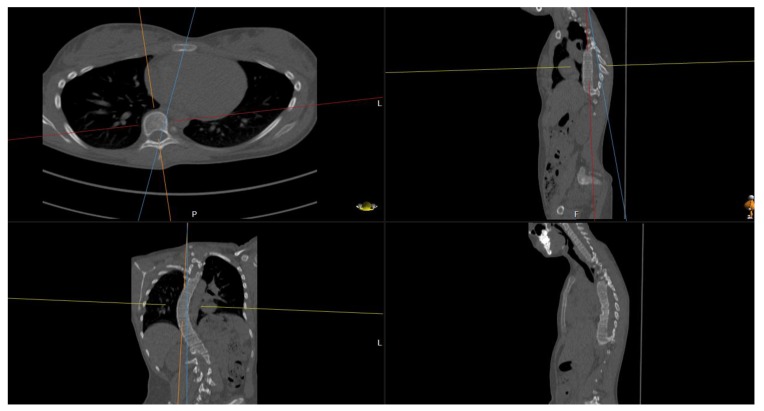
The first step is to estimate an appropriate plane in 3D through the apex.

The method of obtaining the rotation, can be described in the following steps (we have performed the steps manually):

**(1)** Find an appropriate plane in 3D, that passes through an estimation of the center of mass of the apex (see Fig. **2**).

**(2)** Find a line, in the plane from step **(1)**, that passes through the neural groove (in the posterior of the spinal canal) such that the line divides the 2D slice of the vertebral body into two parts roughly symmetric with respect to mass.

**Fig. (3):**
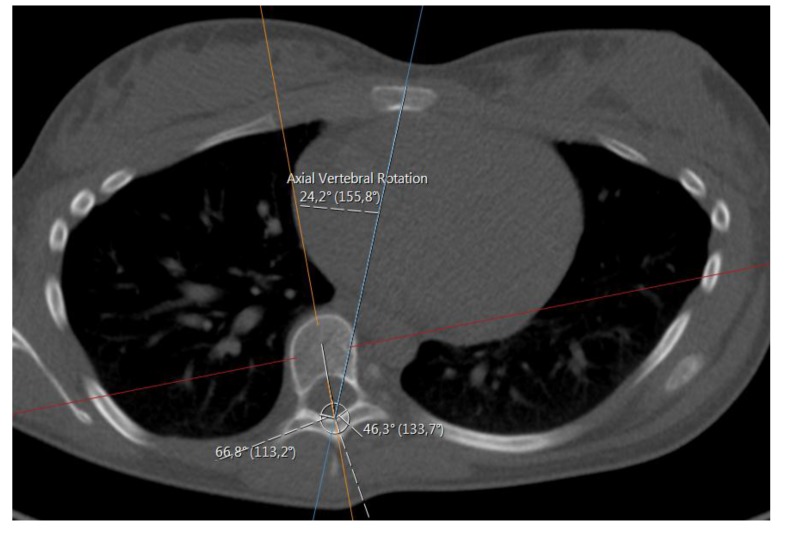
The apical rotation in the sense of Aaro-Dahlborn (with the requirement that the chosen line pass the neural groove), in the text we shall denote it.It is the angle between a line passing through the neural groove and also passing through the exterior mid of the sternum, and a line passing through the neural groove and chosen in the sense of Aaro-Dahlborn (in our case manually and we have after manual choice also verified that a certain symmetry with respect to two equidistant points on the lamina, holds true, see main text).

**(3)** The apical rotation in the sense of Aaro-Dahlborn (with the requirement that the chosen line pass the neural groove), we shall denote it *S_AD_*, is then the angle between the line in step **(2)**, and a line passing through the same neural groove as in step **(2)** and also passing through the exterior mid of the sternum at the level of the plane chosen in step **(1)** (see Fig. **3**). Though our choice of the direction of the line in step **(2)** was made manually, we have a posteriori, verified that our choice is compatible with the following type of symmetry about the neural groove: there is a circle in the plane chosen in step **(1)**, such that the circle is centered at the neural groove in that plane, and defines precisely two points on the interior boundary of the sacral canal, such that the angle at the neural groove, defined by the three points has bisector, that coincides with our chosen line in step **(2)**.

**Fig. (4):**
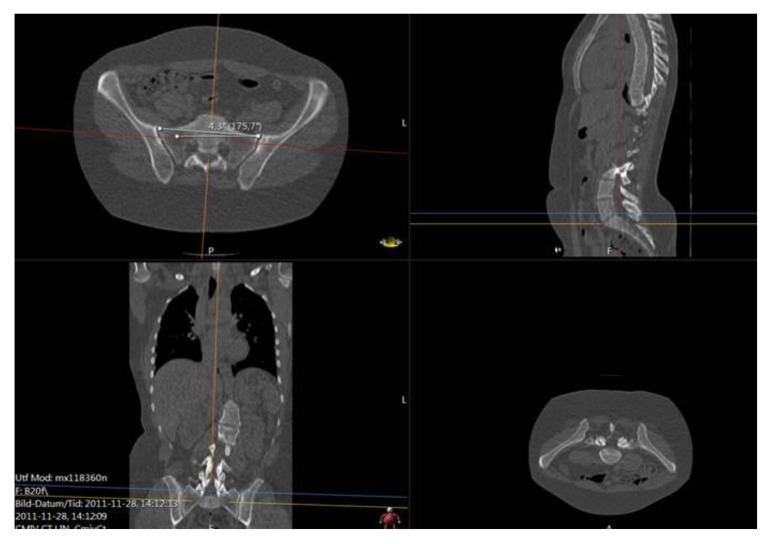
See the text describing steps (i)-(iv). The upper left subfigure shows the achieved angle of the sacrum* versus *the bed, note that it passes through anterosuperiormost part of the sacro-iliac joints, in the given slice. The upper right subfigure shows a plane, which from a sagittal view passes the anterosuperior margin of S1: The blue line in the lower left figure shows the tilting chosen for the sacrum (see main text for more details). The angle representing the tilt and rotation of sacrum (or rather S1) relative to the scanner bed, is the angle between the projection (on an axial plane) of the line in the upper left subfigure (the line depicted between the sacroiliac joints), and the axial horizontal axis.

We shall also use a representative for the tilting and rotation of sacrum (and in some sense the hip), relative to the scanner bed. Here one measures the angle, henceforth called the *sacrum to table angle*, between the axial horizontal with the projection of a line passing through the anterosuperiormost part of the sacro-iliac joints, in a plane which is tilted in line with the tilting of sacrum and the hips (see Fig. **4**) according to the following outline:

**(i).** Move in sagittal view to a plane, parallel to the ’image horizontal’/’bed’, passing through the promontorium sacrum (in the sagittal view this choice is exemplified in the upper right subimage in Fig. (**4**), yellow line). Keep the red line approximately in the center of mass of the projection of sacrum.
**(ii).** In the axial view, set an auxiliary plane (red line in the axial view) passing through the most anterior two points of the two foramina appearing in the view.
**(iii).** Then, fixing the choice of sagittal view described above, go to the coronal view and introduce a second auxiliary plane that it passes through the two valley points of the superior sacral notches (see blue line in lower left subfigure in Fig. **4**).
**(iv).** Finally tilt the first plane through the promontorium, such that it is, in coronal view, parallel to the coronal slice of the second auxiliary plane.
**(v).** The angle used for sacrum/hip-enlignment, called the *sacrum to table angle*, is that which in the axial plane (upper left sub Fig. **4**) is measured, between a line through the frontolateral points of the sacroiliac joints, and a line parallel to the image bed. The line which makes that angle with the bed is called the *sacrum line*.

### 3.3 An Algorithm for Manifesting Artificial, Basic Geometric Model of Scoliosis Vertebrae.

**Fig. (5):**
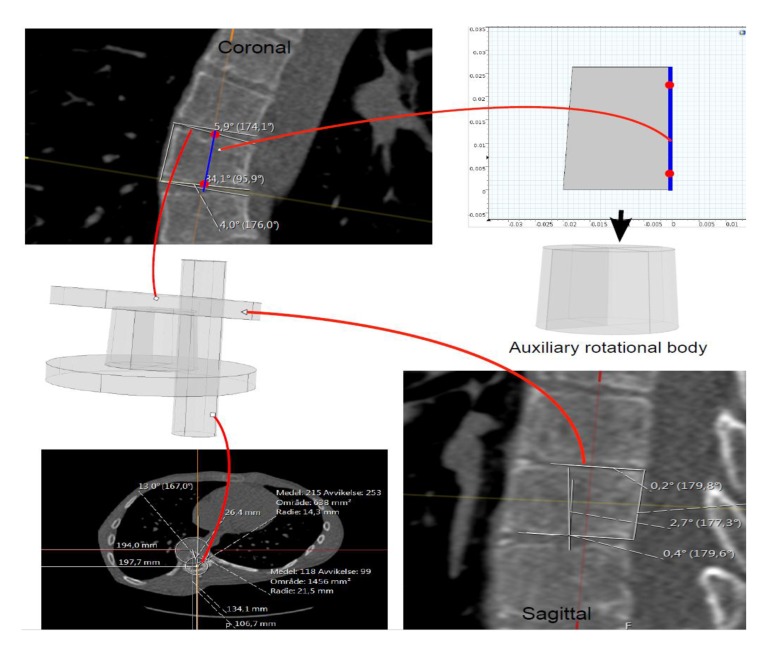
Visual aid for the described steps of the algorithm of the artificial geometric vertebra construction described stepwise in the main text.

The model extraction process from DICOM (Fig. **5**), goes as follows:

#### 3.3.1 Step 1 (Defining a Preliminary Rectangle)

(a) In the environment called MPR (Multi-Plane Reconstruction), in PACS, with the possibility of extracting tilted plane slices from the original DICOM stacks, we fix the sagittal, axial and coronal planes, such that they pass an approximate center of mass of the vertebral body, for the vertebra that we wish to model. The coordinate system will be the same in our Comsol model (*i.e.* based upon the scanner bed).

(b) In the fixed xz-plane we place a central line through the mid points of the upper and lower boundaries of the vertebral body (the line is depicted in the coronal plane in Fig. (**5**), upper left part). The height of the orthogonal projection of the vertebral body, in the slice, on this line, is used as the height of the auxiliary rotation body which we shall define in (c).

(c) Translating this line such that it passes the lower left point of the vertebra in the slice, and then drawing orthogonal lines at the lower left and upper left point of the vertebra in the slice, we obtain three sides of a preliminary rectangle. The upper and lower sides of the vertebra in the slice, yield two angles with respect to the upper and lower parts of the preliminary rectangle (the two angles are depicted in the coronal plane in Fig. (**5**). These angles will be used in Step 2 below for defining wedge properties.

#### 3.3.2 Step 2 (Defining a Trapezoid and Auxiliary Rotational Solid)

(a) We define a trapezoid that whose one side is parallel to a vertical axis (see upper right part of Fig. **5**), The height of our trapezoid will be the height defined in the last step. For the lower horizontal length, we extract a slice (in MPR) approximately parallel to the lower face of the vertebral body in 3D (depicted in upper right part of Fig. **5**), and in that slice we extract the radius of an approximate circle juxtapositioned with the vertebral body in the slice. Similarly, we obtain the upper horizontal length of our trapezoid. (b) Rotating this trapezoid about its right vertical side we obtain a preliminary rotational body depicted directly below the trapezoid in Fig. (**5**).

#### 3.3.3 Step 3 (Accounting for Wedge Properties)

Using the angles that the upper and lower sides of the vertebral body make with the preliminary rotational solid, in the xz and in the yz-planes respectively, we use Boolean operations, with auxiliary cylinders, to manifest the wedge properties in the xz and yz-planes respectively. This is depicted directly below the coronal slice in Fig. (**5**).

#### 3.3.4 Step 4 (Tilting)

We rotate the wedged solid about the x-axis and about the y-axis respectively (it is clear that rotation about any two axes suffices to completely describe a given 3D rotation of any vector), in order to obtain the approximated tilting in 3D that we observe in the MPR (this step will affect the tensor which we need to input in Comsol, regarding material parameters, in the orthotropic case). The rotation angles are measured in the sagittal and coronal planes as depicted in Fig. (**5**). Fig. (**6**) shows a less detailed schematic of the artificial geometric vertebra construction described in the above steps.

### 3.4 Preprocessing Flow Scheme for Manifestation* Via *Segmentation from DICOM.

Using the approximated material parameters of Table **1**, and the simplifications described below, we can summarize the flow chart of going from DICOM stacks to Comsol compatible meshed solid, in Fig. (**7**). FreeCAD/Meshlab), these steps are not included in the flow chart. Using these middle programs we refine, repair and convert to solid and, most importantly, convert the format to a CAD-compatible format, which is then imported into Comsol. There we attempt to mesh it. In most cases, this failed and we had to go back to the middle program for further repair and simplification. Then we also constructed a model that was simplified further using a built-in harmonic smoothing application (rendering smoother and more curved surfaces).

### 3.5 Choice of Load-States

**Fig. (9):**
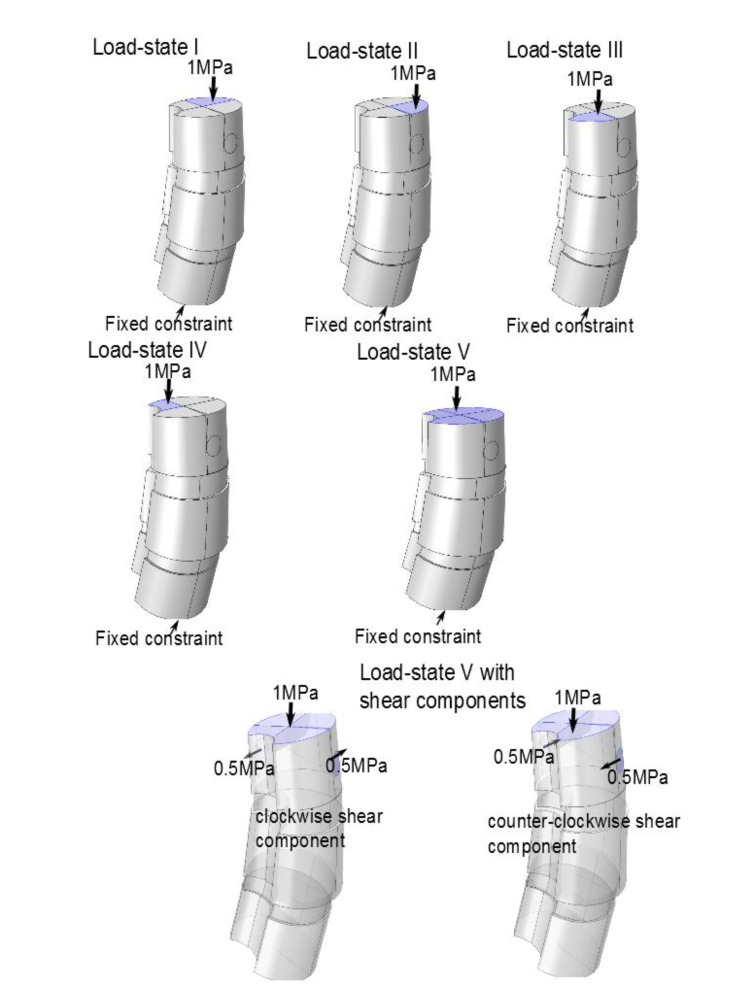
The lower face of the bottommost vertebra was fixed, and we subdivided the upper face of the uppermost vertebral body into four quadrants. In each of the first four load-states (denoted as load-states I-IV), precisely one of the quadrants I-IV, was subdued to downward stress. The fifth load-state is obtained by fixing the lower face of the bottommost vertebra, and applying an evenly distributed stress on the upper portion of the uppermost vertebral body, *i.e.* all four quadrants of Fig. (**8**) are stressed. The additional shear analysis was done by applying shear stress on circular lateral portions, counter-clockwise and clockwise respectively. The lower part of the image illustrates this for load-state V.

We investigated load-states with and without additional applied shear component (in the axial rotational sense), specifically we looked at five different load-states without axial/rotational shear, each corresponding to a reasonable situation which a spine could undergo, and then we used the same settings but with simultaneous shear (axial rotational) stress added to the processus spinosus. The latter shear addition is done both clockwise and counter-clockwise respectively. This yields a total of 5x3=15 load-states. The first four non-shear load-states where set up by fixing the lower portion of the bottom vertebra, and subdividing the upper portion of the uppermost vertebral body, into four parts (in order to do this geometrically and anatomically consistent, we defined a ’horizontal’ line to be a line parallel to the enlignment of the sacrum/hips (Section 3.2), the so called sacrum to table angle gave us this latter line. The subdivision is based upon an approximate circle determined using the ventral part of the vertebral body in the chosen slice (by ventral part we mean up to the ventral junction with the processus spinosus in the given slice). Then translate the horizontal line such that it approximately passes through the center of the above circle for the uppermost vertebra in the model (in an appropriately chosen apical slice) and we automatically obtain a vertical line in the chosen slice by using the perpendicular to our ’horizontal’. This gives us precisely four quadrants, Fig. (**8**). For the five non-shear load-states, the lower face of the bottommost vertebral body was used as fixed constraint, the upper face of the uppermost vertebral body was divided into four quadrants. In each of the first four load-states, precisely one of the quadrants, was subdued to downward stress. The fifth load-state is simply, fixing the lower face of the bottommost vertebra, and applying an evenly distributed stress on the upper portion of the uppermost vertebral body, *i.e.* all four quadrants of Fig. (**8**) are stressed. The additional shear analysis was done by applying shear stress on circular lateral portions, counter-clockwise and clockwise respectively (Fig. **9**).

## 4 RESULTS

Table **1** gives some basic specifications on our patient group and measurement result from the measurements made from the DICOM data using PACS, and Fig. (**10**) gives an illustration of the models. The similar calculations in Table **2** for the states with additional shear and for the orthotropic case where not expected to yield any better linear relations, and we did not overall see statistically significant differences in our t-tests.

### 4.1 Results of Response Angle with Respect to Apex Rotation for the Artificial Geometric Model, the Comparison between Isotropic and Orthotropic Modeling and the Analysis of the Effect of Additional Shear

Table **2** and Figs. (**11**, **12** and **13**) for the apex top response angle results. Table **3** gives the results regarding the analysis of additional shear components in the loads and also the comparison between isotropic and orthotropic modeling.

### 4.2 Method Investigation Results, Involving Segmentation Models and Comparison in Calculation and Meshing Times, with the Artificial Geometric Models

In our method investigation involving segmentation model comparison, we used a HP Elitebook 8540w, with 8Gb RAM. Meshes of type free tetrahedral coarse were used in the mesh-time comparisons analysis. In the test-runs on single apex for the manually segmented model, mesh of type coarser were used, due to a shortage of RAM in our equipment. Even so, it was clear that the calculation times were larger than for the other two methods. It makes sense to analyze the meshing time also separately (even though the simulation time of a test run often includes a meshing process) because first of all, the meshing process is completely independent of whether or not one is modeling isotropically or orthotropically with respect to the material parameters, and second of all, once a mesh is constructed, different boundary and fixed constraints will render different levels of complexity depending on how much of the rough and irregular surfaces are included for the segmentation models. In our test-runs for comparing calculation times, we did not use all three vertebrae and two discs, but rather only the apex. As fixed constraint we fixed the lower part of the vertebral body, and as boundary load we applied 1 MPa in the negative z-axis-direction, to the intersection of the top of the vertebral body, with a cylinder whose radius was adapted to the vertebral body. Fig. (**14**) for the set-up and execution of test-runs for the apex with isotropic material parameters. We performed the test runs with isotropic parameters and orthotropic parameters separately. The meshing times are given in Table **4** (recall that the meshing process is completely independent of whether or not one is modeling isotropically or orthotropically with respect to the material parameters). The calculation times are given in Table **5**. Each of the time values in both tables was reproduced (±1 seconds) three consecutive times.

## 5 DISCUSSION

We view this as a method article, the number of patients is too small to draw statistical conclusions using correlation coefficients, but we do obtain indications on which load-states could be of interest for further experiment set-ups. Also we do not necessarily expect to find linear relations between our response variable and apex rotation but an analytic relation could exist.

The main pros of our method involving artificial geometric models is of course that it enhances the semi-global mechanical responses to applied stresses and that it requires less calculation times due largely to less complicated interfaces. The main cons are that local details are lost in the process (which for example should be present in the mechanical analysis of screws and rods interacting with the vertebrae or in the study of morphology). Note that the Cobb angle is a parameter that is not local to the apex, indeed the neighbours of the apex and their relation to it, do not necessarily reflect the severity of the total coronal curvature. On the other hand, the apex rotation, as we have used in this paper, is strictly local and confined to the apex itself, and thereby a more logical choice of deviation parameter (for our purposes). Here are some restrictions that are made in the pre-processing and processing stages:

- The segmentation models where reworked in several repairing loops due to the difficulties in obtaining acceptable meshes (without spikes and holes).
- The material parameters are only approximations based upon previous literature. The bony tissues and intervertebral discs are very complicated materials, and there is currently no method of obtaining consistent ascertained constants for stress-strain properties for such complicated materials.
- Three vertebrae (the apex together with the two most adjacent vertebrae) were included in the study models, hence our analysis is semi-local and focuses on the apex.
- No ligaments, tendons, nucleus pulposus or end-plates on discs were directly included in the simulation, the project is based on very basic and simple modeling, which can in the future be developed into a more sophisticated one.
- The calculations times for the segmentation models where done* via *Wi-fi license, in order to be able to access an important plug-in that enables compatibility with certain CAD features. This means that there is a possibility that if the internet reception would vary in intensity, then so might the calculation times (however we noted carefully at each run, that the indicator for the connection showed full spikes).

For mechanical analysis of manual segmentation models, we would need to adjust or replace our response variable, because the local irregularities should give false peaks if one only looks at slices of the original 3D-data. It would then possibly be more appropriate to extract some thin volume which one subdivides and performs integration of distortion energy over the elements obtained by the subdivision.

We expected, due to our choice of variable, that additional shear stresses would not render a linear relation between our response variable and apex rotation, as opposed to strictly transversal (rather vertical) stresses. This was verified by pairwise t-tests. The reason additional shears were of less interest is due to the lack of explicit surrounding tissue and ligaments in our model. However, we had no prior expectation on whether or not there would be a statistically significant difference between isotropic and orthotropic modeling (which it turned out in general not to be the case). Obviously, the small amount of patient models means that we cannot draw any conclusion about the linearity between our response variable and apex rotation, but we can at best say that we have obtained indications that a correlation could exist between apex rotation and the response variable, for some appropriately chosen load-state, and our results indicate what requisites such a load-state should have.

We note that all our patients had right-convex scoliosis and a slight local lordosis at the apex. The load-state rendering the best correlation coefficient is load-state 2, and it is the only among the 5 basic load-states with the following properties:

(1) In the coronal plane, the stress counteracts the curvature (in the sense that it lies on the thicker part of the wedged slice of the apex).

(2) In the sagittal plane, the stress counteracts the curvature (in all cases a slight lordosis). If this observation can be reinforced in the future with more substantial evidence, we do not have a good explanation for part (2) of the observation.

## CONCLUSION

We introduce an algorithm for constructing artificial geometric models from CT-scans and thereby a formalized way to obtain models, which enhance and focus on certain mechanical responses which otherwise would be diminished due to small surface variations. Calculation and meshing times appear much shorter. We chose a particular response variable based upon distortion energy distribution, which our pairwise t-tests show is non-trivial, and is foremost sensitive to the type of transversal stresses we are interested in. The reason additional shears were of less interest is due to the lack of explicit surrounding tissue and ligaments in our model. *The method is inappropriate for analyses where the local roughness and irregularities of surfaces are wanted features*. We view this as a method article, the number of patients is too small to draw statistical conclusions using correlation coefficients, but we do obtain indications of *e.g.* which load states are promising candidates with regards to the most common patient specifications. The load-state for which we obtained highest correlation between apex rotation and our chosen response angle, was one where the stress, locally, counteracted the coronal curvature and simultaneously counteracted the sagittal curvature. In general, our model does not render different results for isotropic and orthotropic material modeling respectively. By creating three different types of models for a single patient we illustrate the gain in mechanical simplicity, but a loss in detail using our artificial models. We have pointed out in the article that, if we would like to perform analogous analysis on manually segmentation models in future work, an integration process with appropriately chosen volume, at the interesting faces of the apex, and subdivision of that volume, could be one possible way to substitute the apex top response angle.

## Figures and Tables

**Fig. (1):**
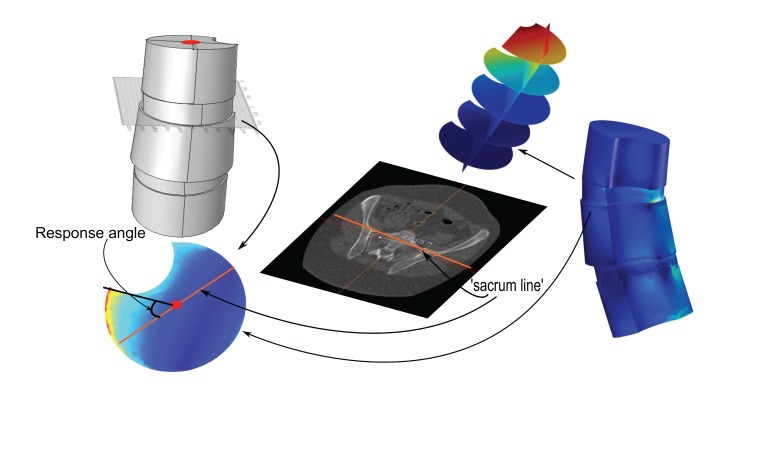
Illustration of how to obtain the so called apex top response angle. Note that the very definition requires a given model (including a certain line describing the tilting and rotation of the hips relative to the scanner bed) extracted from CT, together with a given load-state for that model. This implies that one can calculate pointwise distortion energies (see the rightmost image, showing the result of such calculations for the full solid). Comsol allows for extraction of subdata corresponding to slices in the model (examples of such slices are depicted above the solid response image). For the response variable, we extract a plane parallel to a face of the apex (see lower left image). In this figure, we can find the point corresponding to the approximate relative center of mass (depicted by a red circle) in the slice, and the translation of the sacrum line (Section 3.2 regarding how to find the sacrum line) that passes that point. The apex top response angle is the angle at the aforementioned point, between the sacrum line and a line passing through the focus point, usually, this is a unique point, but in cases that it is not and where artifacts can be suspected, ad hoc choice by the investigator was used.

**Fig. (6):**
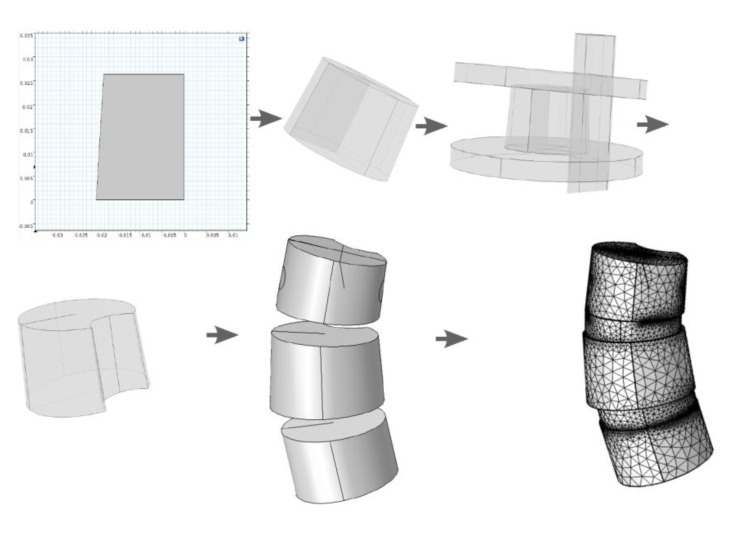
Overview schematic of the artificial geometric vertebra construction.

**Fig. (7):**
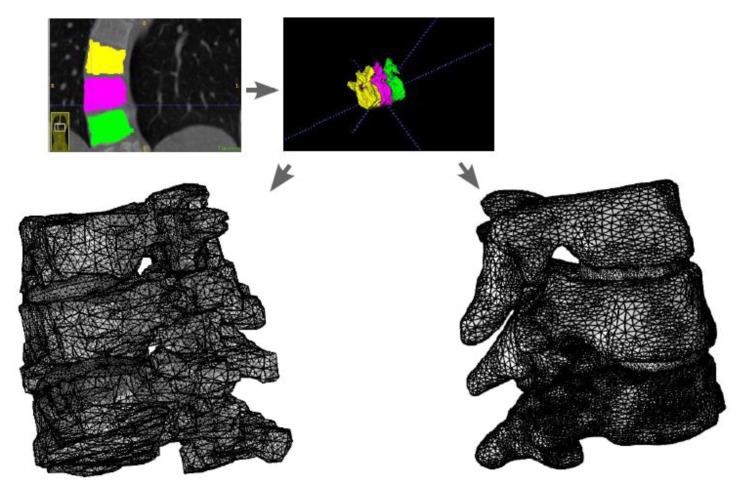
We choose to segment initially in ITK-snap. From there we export STL-formats into several middle programs (mainly CAD-based/compatible programs such as FreeCAD/Meshlab), these steps are not included in the flow chart.In the lower left we see a direct manual segmentation. We also constructed (lower right) a model that was simplified further using a built-in harmonic smoothing application.

**Fig. (8):**
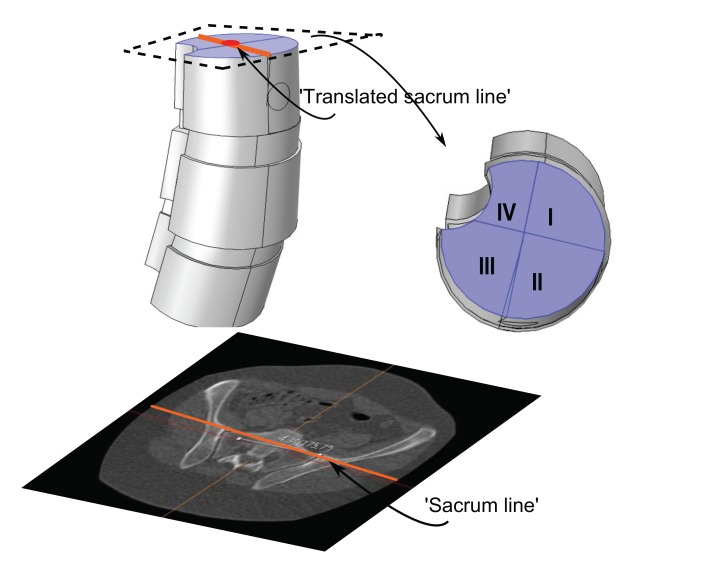
Illustration of how the sacrum to table angle was used to obtain four quadrants, which were in turn used to define the load-states.

**Fig. (10):**
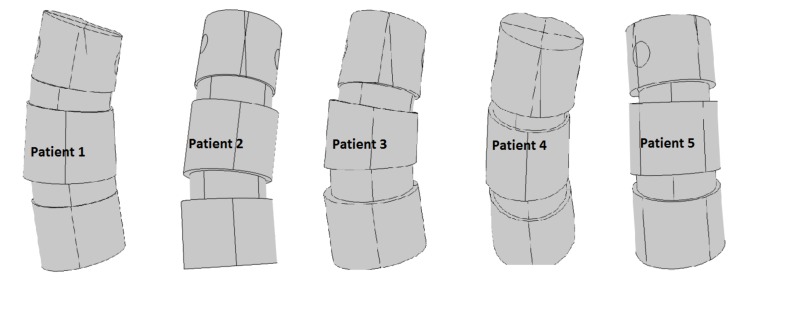
Illustration of the underlying models without applied stresses.

**Fig. (11):**
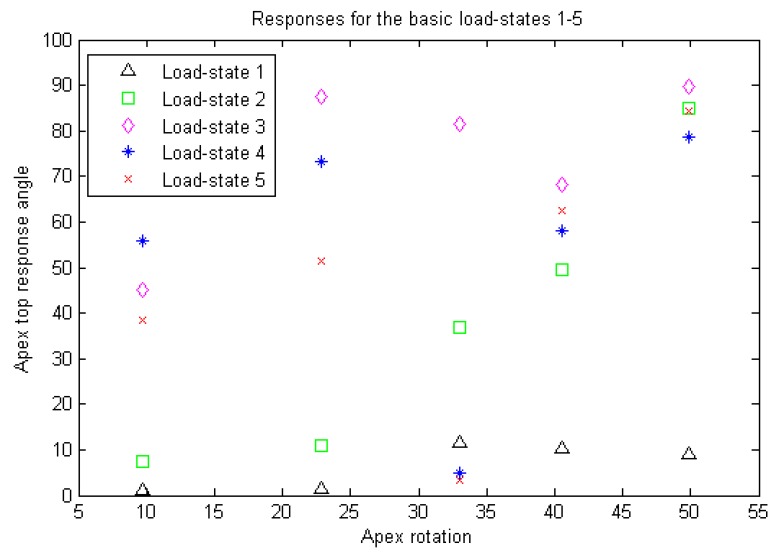
Apex top response angle with respect to apex rotation for the basic load states 1-5.

**Fig. (12):**
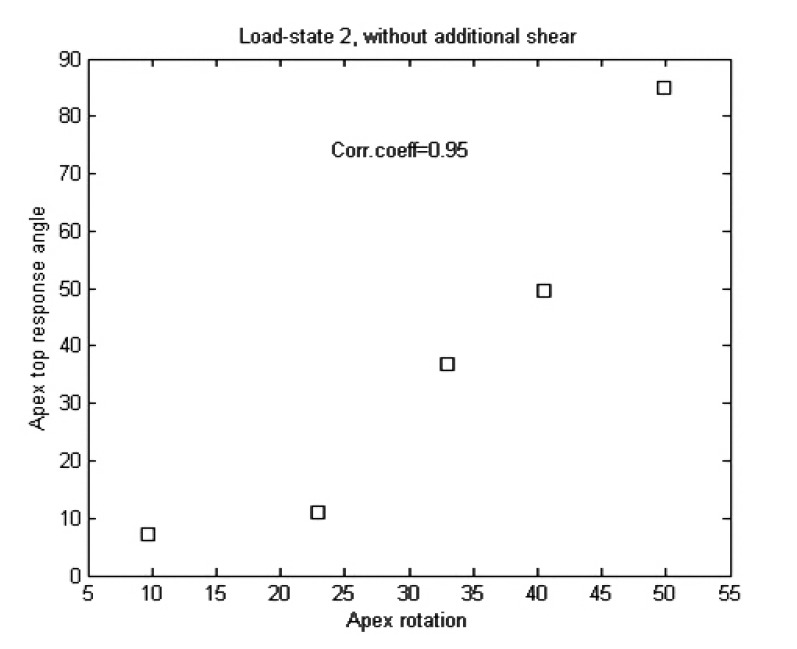
The load state with highest correlation coefficient

**Fig. (13):**
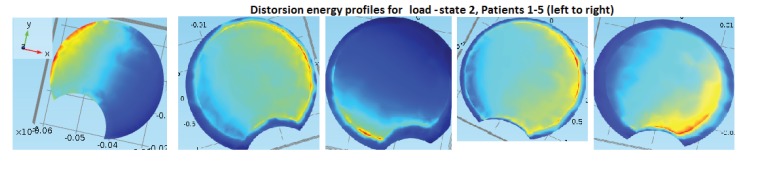
Illustration of distortion energy profiles associated to *Figure 12*

**Fig. (14):**
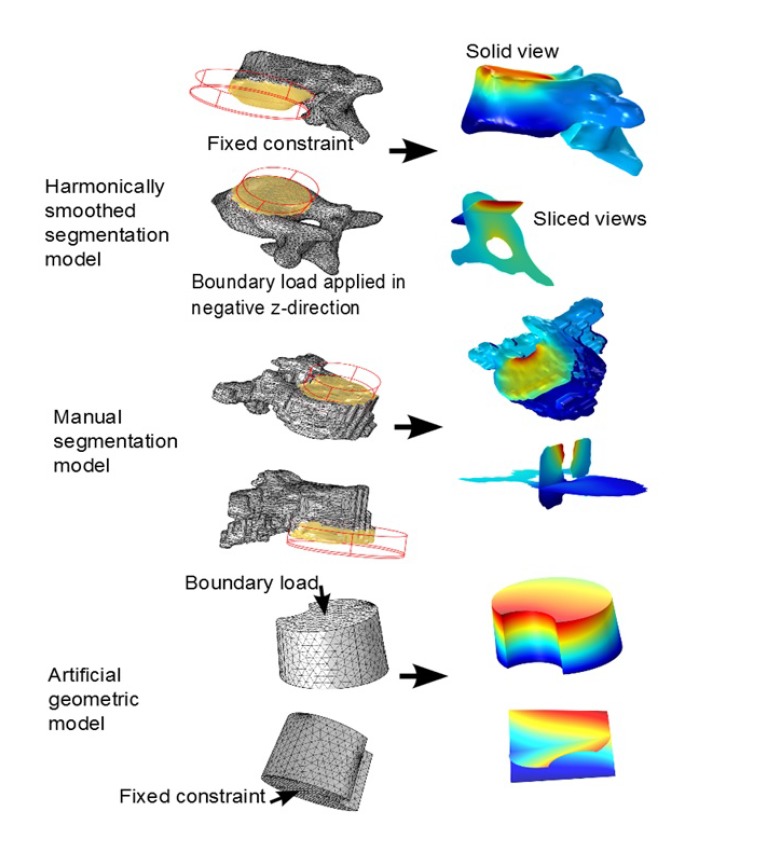
The figure depicts the set-up and execution of test runs with isotropic parameters, for the ordinary manual segmentation model, the segmentation model using harmonic smoothing and the artificial geometric model respectively. The test load is 0.05 MPa and applied in the negative z-direction, on the intersection of the top face of the model, with a given cylinder. The fixed constraint is the intersection of the lower part the vertebrae with a user-defined lower cylinder. This type of test run will process faster than if we were to include 3 vertebrae and discs but will suffice to make clear whether or not a difference between isotropic and orthotropic modeling will render practically tangible different calculation times, and also whether or not the gap between meshing times compared to artificial geometric modeling applies also for calculation times. The result of the test run is displayed for the surface and also using slice planes.

**Table 1:** Patient specifications. The sacrum to table angle is written as positive if the sacrum line when view from above along the z-axis, is rotated clockwise. All patients have primary right-convex thorakal cobb-curve, *i.e.* the curvature in the coronal plane is right convex. The age is that at pre-op CT.

Patient	Convexity	Diagnosis subtype	Apex level	Cobb angle	Apex rotation	Sacrum to table angle	Age
1	Right-convex	Idiopathic	T9	47	33	-1.5	17
2	Right-convex	Idiopathic	T7	46	9.7	7.3	18
3	Right-convex	Idiopathic	T10	82	49.9	0.4	15
4	Right-convex	Idiopathic	T8	50	22.9	2.1	16
5	Right-convex	Idiopathic	T9	18	40.5	2.7	14

**Table 2:** Correlation coefficients between the apex rotation and apex top response angles for the 5 basic load-states.

Load state	1	2	3	4	5
Corr.coeficcient	0.79	0.95	0.67	0.11	0.49

**Table 3:** Statistical pairwise t-tests for difference in apex top response angle, for variations of the models using the basic 5 load-states. Based upon the mean of the variable obtained by subtracting the stochastic variable corresponding to the first type of model, from that corresponding to the second type of model (the Matlab built-in function ’ttest’ was used).

Isotropic models, basic states* versus *with additional clockwise shear components	n=25, p-value ≈ 0.73
Isotropic, basic states* versus *with additional counter-clockwise shear	n=25, p-value ≈ 0.79
Isotropic, with additional counterclockwise versus clockwise shear	n=25, p-value ≈ 0.97
Isotropic* versus *orthotropic models	n=25, p-value ≈ 0.084

**Table 4:** Comparison of meshing times for the apex in three different models.

–	Lower Vertebra	Apex	Upper Vertebra
Harmonically smoothed model	38 sec	25 sec	13 sec
Manually segmented model	39 sec	37 sec	15 sec
Artificial geometric model	<0.5 sec	<0.5 sec	<0.5 sec

**Table 5:** Comparison of calculation times for test runs on the apex in three different models, using isotropic parameters and ortotropic parameters respectively. Due to a shortage of RAM in our equipment, the test-runs for the manually segmented model were made with a mesh of type coarser, whereas the others used type coarse. Each of the values was reproduced ±2 second three consecutive times.

–	Orthotropic	Isotropic
Harmonically smoothed model	31 sec	30 sec
Manually segmented model	49 sec	49 sec
Artificial geometric model	5 sec	5 sec

**Table A1:** Some material constant approximations based upon Kurutz [18], Schmidt *et al*. [24] and the references therein. *Y* denotes the (determining elements of the matrix of) Young modulus (elastic moduli) in MPa, and *v* the Poisson ratio.

**The orthotropic model**	$\left[\begin{array}{c} Y_{\mathrm{xx}}\\ Y_{\mathrm{yy}}\\ Y_{\mathrm{zz}}\\ Y_{\mathrm{xy}}\\ Y_{\mathrm{yz}}\\ Y_{\mathrm{xz}} \end{array}\right]=\left[\begin{array}{c} 11300\\ 11300\\ 22000\\ 3800\\ 5400\\ 5400 \end{array}\right],\left[\begin{array}{c} V_{\mathrm{xy}}\\ V_{\mathrm{yz}}\\ V_{\mathrm{zx}} \end{array}\right]=\left[\begin{array}{c} 0.484\\ 0.203\\ 0.203 \end{array}\right]$
**The orthotropic model**	Y=3500, ν=0.25, Density^1^≈1908 kg/m^3^
**Intervertebral disc^2^**	Y≈252, ν≈0.47, Density^3^≈1120 kg/m^3^

^1^Built-in approximation, see main text. ^2^ Based upon Kurutz [18] p.219-223, elastic fiber approximation (Y = 500,ν= 0.45) and fluid-like solid for nucleus pulposus (Y = 4, ν = 0.50), mean value was used. ^3^Approximated as 60% water and 40% collagen, see main text.

**Table A2:** Voigt map to the index pairs (i,j) and (n,m)s

	η(ij)=1	η(ij)=2	η(ij)=3	η(ij)=4	η(ij)=5	η(ij)=6
η(ij)=1	1	0	0	0	0	0
η(ij)=2	0	1	0	0	0	0
η(ij)=3	0	0	1	0	0	0
η(ij)=4	0	0	0	½	0	0
η(ij)=5	0	0	0	0	½	0
η(ij)=6	0	0	0	0	0	½

## APPENDIX

### A.1 Distortion Energy

The failure theory of von Mises, based upon maximum distortion energy, roughly assumes that some notion of ’failure’, of a medium under stress, occurs when the maximum distortion energy at a reference point, in a stressed material reaches the value of the maximum distortion energy at the failure point in simple uniaxial tension (this failure stress is denoted by *σ_failure tensile_*), and the distortion energy is given by the left hand side of the following equation,

(A1) $$\sigma_{1}^{2}+\sigma_{2}^{2}+\sigma_{3}^{2}-(\sigma_{1}\sigma_{2}+\sigma_{2}\sigma_{3}+\sigma_{1}\sigma_{3})=2\sigma_{\mathrm{failure},\mathrm{tensile}}^{2}$$

Where *σ_i_*, *i* = 1, 2, 3, denote the principal stresses. Akin [14], p.267, defines the von Mises stress as the square root of, one half of the left hand side of equation 2, namely,

(A2) $$\frac{1}{\sqrt{2}}\sqrt{(\sigma_{1}-\sigma_{2}{)}^{2}+(\sigma_{2}-\sigma_{3}{)}^{2}+(\sigma_{3}-\sigma_{1}{)}^{2}}$$

and relates the von Mises stress to the distortion energy. Although the von Mises energy definition was constructed based on the isotropic case, one can use the same definition for any case when the principle stresses are calculated, and it is then an approximative entity for describing distortion energy matters.

### A.2 Material Parameters

For the Comsol model one must have certain physical constants and parameters (or approximations of them) at hand. Bones and other human tissue are very complex materials chemically and physically. Bone is in general a connective tissue consisting of cells embedded in a mineralized matrix including collagen fibers which are impregnated with calcium phosphate mainly as hydroxyapatite, as well as carbonate, citrate, sodium, and magnesium. Bone is in general composed of about 75% inorganic material and 25% organic material. Different types of bones vary in physical properties, *e.g.* the diaphyses of the tibia and femur have a rather large marrow, and a complicated Haverian system of canals, whereas such features are much less prominent in *e.g.* the frontal cranium. One of the features of certain bones, such as diaphyseal bones and also to smaller extent, the vertebral bodies that encourages an orthotropic modeling, is the fact that osteones have concentric lamellae that induce an intrinsic main/longitudinal axis. The densities of bone and vertebral discs are sometimes required inputs for defining a material in FEM models, and in such case we have used 1908 kg/m3 (the software built-in value of density of generalized bone). For the intervertebral discs, we use, following Chen & Thouas [15], p.400, the density of dry collagen to be approximately 1300 kg/m3, and we approximate the density equivalent composition as 60% water and 40% collagen, (in fact, both the composition for the annulus fibrosus and nucleus pulposus contain also proteoglycan and third main component, thus we have made an approximation of using the dry weight of collagen to approximate that of proteoglycan). Even after approximations regarding mechanical and physical properties there remains other complications such as age and gender, see Ebbesen *et al*. [16]. We mention some research articles including material constant estimation, see *e.g.* Antosik *et al*. [17], Kurutz *et al*. [18] and Nédli *et al*. [19]. Other sources in which there appear material constant estimates are Keaveny *et al*. [20], Ch.8, Currey [21] and Frankel & Nordin [22]. Fracture stress of a general bone has in some contexts been estimated to be 14±25% (MPa), see Vable [23], p.3.

However it is commonly known that bones behave anisotropically and under tension, and viscoelastically (*i.e.* the stress-strain curves depend upon the rate of load application), see *e.g.* Frankel & Nordin [22]. The primary sources however, used in the collecting of material constants and insight to the material properties for us have been: White [25], Kurutz [18], Wilke *et al*. [26], Stemper *et al*. [27], Schmidt *et al*. [24], Ambrosio & Tanner [28] (and indirectly Brown, Hansen and Yorra [29]), and the references found in these later articles. Interpretation of such data requires a prescribed main axis (in the case of our cited articles, the z-axis) in the coordinate system for which the data holds true, and a basic knowledge of the anatomy and structure of bone makes it clear that the main axis can, for the epiphyseal case, be chosen along the Haverian longitudinal axis, and for vertebra, as one passing through the center of the vertebral body and approximately tangential to the spinal cord. We have chosen to mainly use the constants gathered from different literature in Schmidt *et al*. [24]. Note that such approximations impose the difficulty that osteoporosis, if not taken into account, may distort the authenticity of the simulation.

### A.3 Assigning Material Properties for the Orthotropic Models, in Practice

The feature we use in Comsol, starts with applied force, applied loads and fixed constraints. The output is a version of distortion energy at each point (we use stationary analysis, as opposed to time-dependent). The calculations performed by Comsol thus requires the input of material parameters in terms of either compliance or elasticity components. Assuming *linearly elasticity*, there exists a tensor (see *e.g.* Irgens [30]), *S*, let us denote its components by *s*_ijkl,_ interpreted as a fourth order tensor, which acts on strain tensors (ε, which are second order tensors in their own right) and yields the associated stress tensor (σ, also a tensor of second order) and is sometimes referred to as the elasticity tensor. The *elasticity tensor* is the inverse (in the appropriate sense) of the so called compliance tensor, let us denote it *C*. Both the strain and stress tensors are symmetric, thus each is completely determined by 6 of its components (namely σ_ij_ = σ_ji_ for i≠j, so we only need to know the upper triangle part of a matrix representation of σ), and we can represent *C* by 36 components in matrix form, using the so-called *Voigt map* of indices:

(A3) η:{11,22,33,23,32,13,31,12,21}→{1,2,3,4,5,6}

(A4) η(11)=1,η(22)=2,η(33)=3

(A5) η(23)=η(32)=4,η(13)=η(31)=5,η(12)=η(21)=6

which induces,

(A6) $$C_{\mathrm{ijkl}}\mapsto C_{\eta (\mathrm{ij}),\eta (\mathrm{kl})}$$

The tensors *C* (and analogously *S*) can be shown (see *e.g.* Kittel [31] or Zehnder [32], p.15) to be symmetric in the sense that,

(A7) $$C_{\mathrm{ijkl}}=C_{\mathrm{jikl}}=C_{\mathrm{ijlk}}=C_{\mathrm{klij}}$$

The relation between the arrays *S* and *C* can be given in tensor form, is given by (see *e.g.* Barnett *et al*. [33], p.15),

(A8) $$C_{\mathrm{ijkl}}S_{\mathrm{klmn}}=\frac{1}{2}(\delta_{\mathrm{im}}\delta_{\mathrm{jn}}+\delta_{\mathrm{in}}\delta_{\mathrm{jm}})$$

where δ_ij_ for i=j, and zero otherwise. The right hand side of the last equation has a 6x6 representation, when applying the Voigt map to the index pairs (i,j) and (n,m).

Let η_1_,η_2_,η_3_, be indices obtained by applied the Voigt map to the index pairs (i,j), (k,l) and (n,m) respectively, and *C_η1η2_*, *S_η1η2_* let denote the tensor components after having transformed the indices. Then the left hand side of equation (A8) can (using the symmetry relations of equation (A7)) be described as,

(A9) $$\sum_{\eta_{2}=1}^{3}C_{\eta_{1}\eta_{2}}S_{\eta_{21}\eta_{3}}+2\sum_{\eta_{2}=4}^{6}C_{\eta_{1}\eta_{2}}S_{\eta_{21}\eta_{3}}$$

Combining the last two representations we obtain 36 equations which can be represented in matrix form according to (note that some text books prefer to use c for the elements of the elasticity tensor, we have chosen to use c for the elements of the compliance tensor),

$\overset{\underset{︸}{\left[\begin{array}{cccccc} S_{11} & S_{12} & S_{13} & S_{14} & S_{15} & S_{16}\\ S_{21} & S_{22} & S_{23} & S_{24} & S_{25} & S_{26}\\ S_{31} & S_{32} & S_{33} & S_{34} & S_{35} & S_{36}\\ S_{41} & S_{42} & S_{43} & S_{44} & S_{45} & S_{46}\\ S_{51} & S_{52} & S_{53} & S_{54} & S_{55} & S_{56}\\ S_{61} & S_{62} & S_{63} & S_{64} & S_{65} & S_{66} \end{array}\right]}}{=:S^{\mathrm{voigt}}}\overset{\underset{︸}{\left[\begin{array}{cccccc} C_{11} & C_{12} & C_{13} & {\mathrm{2C}}_{14} & {\mathrm{2C}}_{15} & {\mathrm{2C}}_{16}\\ C_{21} & C_{22} & C_{23} & {\mathrm{2C}}_{24} & {\mathrm{2C}}_{25} & {\mathrm{2C}}_{26}\\ C_{31} & C_{32} & C_{33} & {\mathrm{2C}}_{34} & {\mathrm{2C}}_{35} & {\mathrm{2C}}_{36}\\ C_{41} & C_{42} & C_{43} & {\mathrm{2C}}_{44} & {\mathrm{2C}}_{45} & {\mathrm{2C}}_{46}\\ C_{51} & C_{52} & C_{53} & {\mathrm{2C}}_{54} & {\mathrm{2C}}_{55} & {\mathrm{2C}}_{56}\\ C_{61} & C_{62} & C_{63} & {\mathrm{2C}}_{64} & {\mathrm{2C}}_{65} & {\mathrm{2C}}_{66} \end{array}\right]}=}{=:C_{0}}\overset{\underset{︸}{\left[\begin{array}{cccccc} 1 & 0 & 0 & 0 & 0 & 0\\ 0 & 1 & 0 & 0 & 0 & 0\\ 0 & 0 & 1 & 0 & 0 & 0\\ 0 & 0 & 0 & 1/2 & 0 & 0\\ 0 & 0 & 0 & 0 & 1/2 & 0\\ 0 & 0 & 0 & 0 & 0 & 1/2 \end{array}\right]}}{G}$

Which is equivalent to,

$\begin{array}{l} \underset{︸}{S^{\mathrm{voigt}}\left[\begin{array}{cccccc} c_{11} & c_{12} & c_{13} & 2c_{14} & 2c_{15} & 2c_{16}\\ c_{21} & c_{22} & c_{23} & 2c_{24} & 2c_{25} & 2c_{26}\\ c_{31} & c_{32} & c_{33} & 2c_{34} & 2c_{35} & 2c_{36}\\ c_{41} & c_{42} & c_{43} & 2c_{44} & 2c_{45} & 2c_{46}\\ c_{51} & c_{52} & c_{53} & 2c_{54} & 2c_{55} & 2c_{56}\\ c_{61} & c_{62} & c_{63} & 2c_{64} & 2c_{65} & 2c_{66} \end{array}\right]==id_{6\times 6}}\\ \;=C_{0}G^{-1}=:C^{\mathrm{voigt}} \end{array}$

In other words, as square matrices *C^voigt^*, *S^voigt^*, satisfy, (*C^voigt^*)^-1^ = *S^voigt^* For an orthotropic material, we can (given the Young/shear moduli and Poisson ratios: *Y_xx_*, *Y_yy_*, *Y_zz_*, *Y_yz_*, *Y_xz_*, *Y_xy_*, *V_xy_*, *V_yz_*, *V_xz_* for appropriate choice of basis, write the compliance matrix, in Voigt notation as,

$C^{\mathrm{voigt}}:=\left[\begin{array}{cccccc} \frac{1}{Y_{\mathrm{xx}}} & -\frac{v_{\mathrm{yx}}}{Y_{\mathrm{yy}}} & -\frac{v_{\mathrm{zx}}}{Y_{\mathrm{zz}}} & 0 & 0 & 0\\ -\frac{v_{\mathrm{xy}}}{Y_{\mathrm{xx}}} & \frac{1}{Y_{\mathrm{yy}}} & -\frac{v_{\mathrm{xy}}}{Y_{\mathrm{xx}}} & 0 & 0 & 0\\ -\frac{v_{\mathrm{xx}}}{Y_{\mathrm{xx}}} & -\frac{v_{\mathrm{yz}}}{Y_{\mathrm{yy}}} & \frac{1}{Y_{\mathrm{zz}}} & 0 & 0 & 0\\ 0 & 0 & 0 & \frac{1}{Y_{\mathrm{yz}}} & 0 & 0\\ 0 & 0 & 0 & 0 & \frac{1}{Y_{\mathrm{xz}}} & 0\\ 0 & 0 & 0 & 0 & 0 & \frac{1}{Y_{\mathrm{xy}}} \end{array}\right]$

which will be symmetric. In our case, based upon the references we used, we specifically consider the case *Y_xx_* = *Y_yy_*, *Y_yz_*, *Y_xz_* (such a model is sometimes called transversely orthotropic) and also *V_yz_* = *V_xz_*. Note that, by symmetry, *V_xy_* = *V_yx_*, *V_zy_* = *V_zx_* and (again in the special case within which we work) If *C* = [*C_ijkl_*] is the compliance array (34 elements), we can for a given rotation in 3D, given by say the matrix R; use the relation (see *e.g.* Irgens [29], p.88),

(A10) $${\overset{\wedge }{C}}_{\mathrm{pqrs}}=\sum_{i,j,k,l}R_{\mathrm{pi}}R_{\mathrm{qj}}R_{\mathrm{rk}}R_{\mathrm{sl}}c_{\mathrm{ijkl}}$$

where $\overset{\wedge }{C}=[{\overset{\wedge }{C}}_{\mathrm{pqrs}}]$ can be interpreted as the compliance matrix of an orthotropic material with respect to the new basis *R*(*e_1_, e_2_, e_3_*)^T^ We can then use Voigt notation to extract the compliance and elasticity array for a ’tilted’ orthotropic material using Comsol. As an example, rotation about the x-axis of *Ø_1_* degrees and rotation about the y-axis of *Ø_2_* degrees respectively are given (if we choose a setting where x increases to the right, y points out of the screen and z increases upward, thereby the following matrices will for positive angles give clockwise rotation, when the axis of rotation points toward observer) by the orthogonal transformation matrices,

$R^{x,\phi_{1}}=\left[\begin{array}{c} 1\\ 0\\ 0 \end{array}\begin{array}{c} 0\\ \cos(\phi_{1})\\ \sin(\phi_{1}) \end{array}\begin{array}{c} 0\\ -\sin(\phi_{1})\\ \cos(\phi_{1}) \end{array}\right],R^{y,\phi_{2}}=\left[\begin{array}{c} \cos(\phi_{2})\\ 0\\ -\sin(\phi_{2}) \end{array}\begin{array}{c} 0\\ 1\\ 0 \end{array}\begin{array}{c} \sin(\phi_{2})\\ 0\\ \cos(\phi_{2}) \end{array}\right]$

The combined transformation matrix becomes, *R* = *R^x, Ø1^**R^y, Ø2^* Using the above formulae, a Matlab code was written in order to calculate the input elasticity matrix elements for tilted vertebrae in the orthotropic models.
